# Viral effectors trigger innate immunity across the tree of life

**DOI:** 10.1098/rstb.2024.0077

**Published:** 2025-09-04

**Authors:** Kevin Barthes, Francois Rousset, Tanita Wein

**Affiliations:** ^1^ CIRI, Centre International de Recherche en Infectiologie, Université de Lyon, INSERM U1111, UCBL1, CNRS UMR5308, ENS de Lyon, Lyon, France; ^2^ Department of Systems Immunology, Weizmann Institute of Science, Rehovot 7610001, Israel

**Keywords:** host–pathogen interactions, effector-triggered immunity, phages, bacterial immunity, immune evasion, innate immunity, viruses

## Abstract

Viruses are ubiquitous biological entities that exert immense selective pressures on their hosts, driving the evolution of diverse innate immune mechanisms across all domains of life. While innate immunity has historically been studied in eukaryotes, recent discoveries of bacterial defence systems against phages reveal striking parallels between prokaryotic and eukaryotic immunity. A key principle of antiviral defence conserved from bacteria to humans is pattern recognition, where virus-associated molecular patterns trigger immune responses. In addition to pattern recognition, effector-triggered immunity (ETI) involves the detection of pathogen-induced perturbations of host cell pathways. ETI, initially described in plants and later in animals, was recently shown to have parallels in bacterial immunity as well. In this perspective, we explore how viral infections in prokaryotic and eukaryotic cells manipulate comparable host pathways, creating molecular signatures that are recognized by distinct immune systems. By examining the shared features and mechanisms underlying ETI, we illuminate its role as a core principle of host–pathogen interactions across the tree of life.

This article is part of the discussion meeting issue ‘The ecology and evolution of bacterial immune systems’.

## 1. Introduction

Biological systems are inherently vulnerable to pathogens, which inevitably arise to exploit resources for their own gain. As a result, nearly all living cells face the threat of viral infections, with viruses being the most abundant biological entities on the planet. Across evolutionary scales, persistent viral pressure has driven the evolution of a vast array of cell-autonomous defence mechanisms encoded by their host cells, together defining the innate immune system. Although innate immunity has traditionally been studied in animals and plants, recent discoveries have revealed hundreds of bacterial defence systems that protect bacteria against phages, their viral predators [1]. Intriguingly, prokaryotic and eukaryotic immunity share conceptual parallels, and some immune components even emerged in prokaryotes and were conserved in eukaryotes over the course of evolution (Box 1) [2,13,17]. These insights are reshaping our understanding of host–pathogen interactions across all domains of life.

Box 1: **Prokaryotic origins of innate immune mechanisms**

The human innate immune system has long been considered the result of extensive evolutionary innovations in metazoans. This view was proven false upon the recent discovery that essential components of the cell-autonomous innate immune system have ancient evolutionary roots in prokaryotic defence systems that protect bacteria from phages [2]. Those include the cyclic GMP-AMP synthase (cGAS)-stimulator of interferon genes (STING) pathway [3], the RNA interference (RNAi) pathway [4], nucleotide-binding leucine-rich repeat receptors (NLRs) [5,6], antiviral effectors such as viperin [7], SAMHD1-like nucleotide-depleting enzymes [8,9], gasdermin proteins [10], ATP nucleosidases [11] and more [12]. To date, since the human immune system is the most characterized, most studies used this knowledge as a basis to identify novel immune proteins in bacteria. Nonetheless, recent discoveries exploiting bacterial diversity have uncovered novel eukaryotic immune genes and mechanisms [11,13–16]. The discovery of these conserved systems suggests that fundamental immune strategies have been shaped by common selective pressures throughout evolutionary history.

A central paradigm of antiviral innate immunity is that of pattern recognition, in which virus-associated molecular patterns (they may be proteins or nucleic acids) are directly recognized by one of many pattern-recognition receptors (PRRs), triggering their activation and downstream immune signalling [18]. Initially described in animals and plants, pattern recognition was recently shown to be central to bacterial immunity as well, where bacterial defence proteins specifically recognize viral structural components or nucleic acids to trigger an immune response [5,6,19].

A distinct class of immune response, termed effector-triggered immunity (ETI), involves sensing the effect of pathogen infection on the cell [20]. During infection, pathogens typically synthesize a multitude of proteins that serve to manipulate host metabolism, to optimize pathogen replication or to evade immune responses. These virulence factors, also called *effectors*, evolve rapidly and may therefore be unsuitable targets for direct recognition by PRRs. Instead, the host cell senses the molecular effect of these effector proteins on specific pathways or cellular processes in the context of ETI [20]. As a consequence, structurally diverse effectors that act upon a common pathway can be detected by the same immune protein, thereby conferring protection against a wide range of pathogens. The principle of ETI includes the ‘guard hypothesis’, which originated from studies of pathogen-mediated immune stimulation in plants [21]. This concept posits that ‘guard’ proteins act as sentinels by monitoring host cellular components commonly targeted by pathogen effectors, and become activated when their normal function is disrupted or modified. ETI was first described in plants and later in animals, but the molecular mechanisms involved are complex and less understood compared to pattern -recognition-based mechanisms [22].

Research has mostly focused on effector proteins from pathogenic bacteria and their roles in manipulating host cellular processes and evading immune responses, providing a robust framework for understanding ETI in eukaryotes [20,23]. In contrast, viral effectors remain less understood, despite their central role in modulating host biology during infection. Like other pathogens, phages also produce a range of effector proteins to manipulate their bacterial host during infection, and their effect on the bacterial cell is susceptible to trigger an ETI-like response [24]. The recent advances in the field of bacterial immunity now place ETI as a previously overlooked aspect of the conservation of immunity across domains of life. In this perspective, we delineate how prokaryotic and eukaryotic viruses can generate comparable molecular signatures on their host, and how these signatures can be sensed in the context of ETI across domains of life. By focusing on viral effectors, we aim to highlight the underexplored aspects of host–pathogen interactions that bridge the domains of life, emphasizing the evolutionary continuity of immune strategies.

## 2. Viral effectors suppressing host transcription

Viruses typically encode transcriptional regulatory proteins that are critical for controlling viral and host gene expression. The precise biological function of such interference is not always fully understood, but is generally assumed to favour viral over host gene expression towards optimizing metabolic resources for the production of viral proteins. Prokaryotes and eukaryotes evolved immune strategies to indirectly sense this interference.

**Figure 1.**
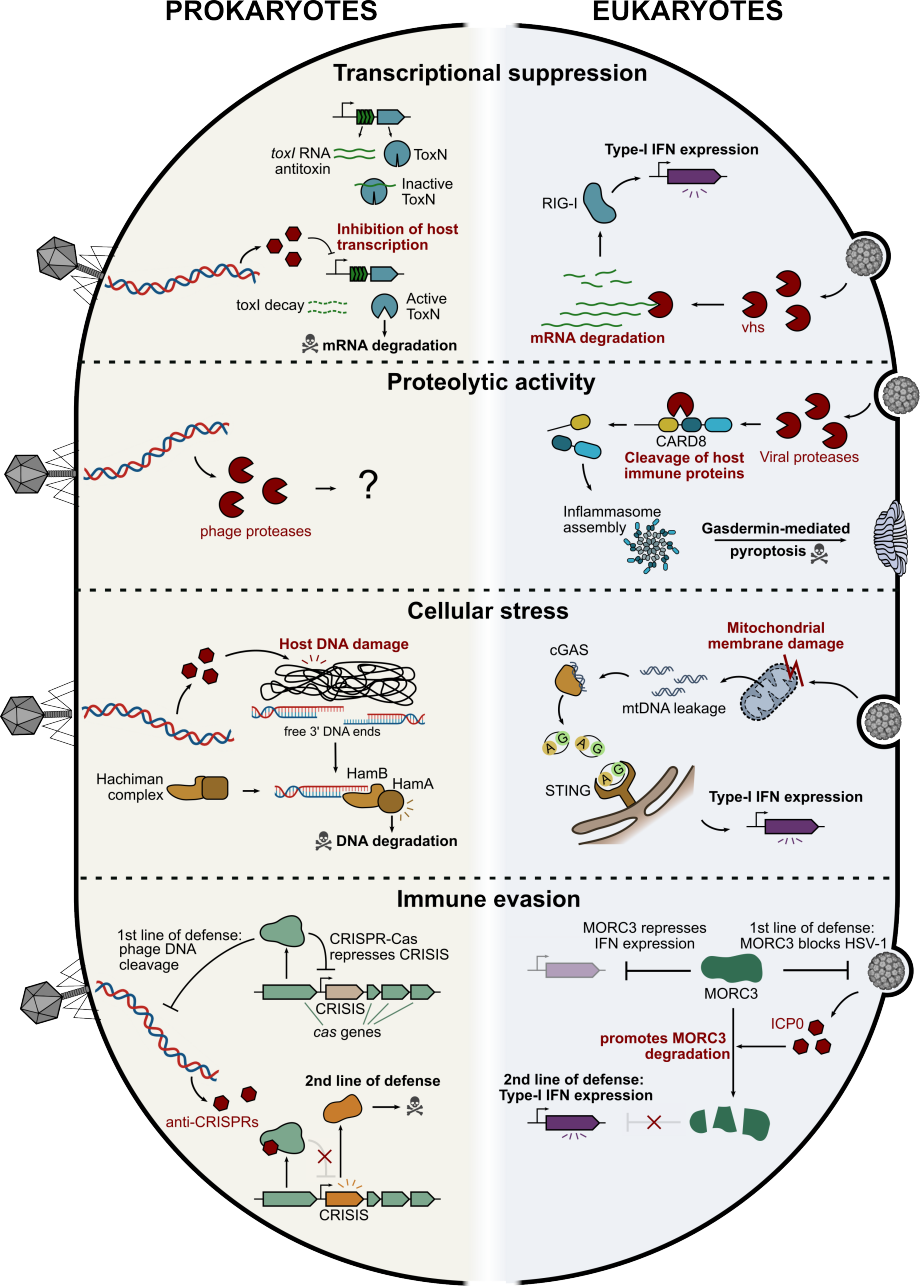
Viral effectors trigger innate immunity through similar molecular signatures in prokaryotes and eukaryotes. Shown are examples of immune responses induced by viral effectors (shown in red) suppressing transcription, cleaving proteins, inducing cellular stress or enabling immune evasion. CARD8, caspase recruitment domain protein 8; cGAS, cyclic GMP-AMP synthase; CRISIS, CRISPR-suppressed immune system; IFN, interferons; mtDNA, mitochondrial DNA; RIG-I, retinoic acid-inducible gene I; STING, stimulator of interferon genes.

Transcriptional regulatory proteins have been identified in multiple families of human viruses, including both DNA and RNA viruses. Transcriptional effectors can alter human gene expression at multiple levels, including mRNA degradation, chromatin organization, RNA polymerase II recruitment, transcription initiation and transcription elongation [25–27]. A well-studied example is the virion host shutoff (vhs) protein that is an RNase encoded by the herpes simplex virus UL41 gene [28]. To access its target RNAs, the RNase associates with eukaryotic translation initiation factors, which bind capped mRNAs. Consequently, vhs specifically degrades fully processed mRNAs in the cytoplasm. Degradation and unmasking of mRNA products by vhs was proposed to be sensed by the retinoic acid-inducible gene I (RIG-I)-like receptor, which leads to the transcriptional induction of type I interferons and other genes that establish an antiviral host response (figure 1) [29,30]. Yet, the exact sensing mechanism is not fully understood.

Like human viruses, multiple phages encode early proteins that disrupt host gene expression, for instance, by interfering with the host RNA polymerase through direct binding or phosphorylation [31–34]. Diverse defence systems, especially toxin–antitoxin (TA) systems, have evolved to indirectly detect this hallmark. TAs consist of a toxic protein and an antitoxin which, under basal conditions, prevents toxin expression or activation, or reverts its molecular activity. For instance, in the type I TA systems Hok/Sok, the antisense RNA antitoxin Sok binds to the mRNA of the Hok toxin to inhibit its translation; following host transcription shutoff by phage T4, Sok antitoxin rapidly decays because of its short half-life (<30 s), allowing translation of the Hok toxin mRNA leading to growth arrest [35]. In the type III TA system ToxIN, the RNA antitoxin toxI directly binds to the toxin protein to block its activity; host transcriptional shutoff by T4 leads to the decay of toxI, releasing the ToxN toxin which functions as an RNAse that depletes cellular mRNAs (figure 1) [36]. Another mechanism of host transcription interference is that of phage T7, where the T7 protein gp5.7 inhibits the σS-dependent bacterial RNA polymerase by direct binding. In turn, the host dCTP deaminase, which is safeguarded under uninfected conditions by a small RNA, is unleashed, leading to the cellular depletion of the nucleotide dCTP that is essential for viral DNA replication [8,37]. A similar sensing mechanism has been hypothesized for dGTPases involved in anti-phage defence, which deplete the nucleotide dGTP to block phage replication [8].

## 3. Protease-mediated protein degradation by viral effectors

Proteases are ubiquitous regulators of protein function in all domains of life and human viruses are known to encode diverse proteases which often participate in the maturation of viral proteins [38,39]. Examples include the HIV protease, as well as coronavirus 3 CL and picornavirus 3C proteases that directly cleave immune proteins like the human caspase recruitment domain-containing protein 8 (CARD8) [40,41]. CARD8 is an inflammasome-forming cytosolic protein complex that acts as an immune sensor. It contains a disordered N-terminus, a function-to-find domain (FIIND) and a caspase recruitment domain (CARD). In resting cells, the FIIND domain is constitutively autoproteolytically processed, while the two resulting fragments remain associated [42,43]. Upon infection, viral proteases degrade the N-terminus, leading to the release of the CARD-containing fragment. By being processed by viral proteases, CARD8 senses viral protease activity, which leads to inflammasome formation, gasdermin activation and pyroptosis, together mounting a systemic immune response (figure 1) [41]. In a similar process, inflammasome formation of human NLRP1 is activated by direct cleavage by viral proteases including HIV, enterovirus 3C and coronavirus 3 CL proteases, resulting in pyroptosis and inflammation [44,45].

While such processes remain unknown for phage–bacteria interactions, phage genomes encode various proteases that play crucial roles in their infectious cycle. For instance, many phages encode capsid assembly proteases that process scaffolding proteins to shape the mature phage capsid [46–48]. The prohead protease from phage Bas13 was shown to interact with the sensor of a CBASS system to induce cellular growth arrest [49], suggesting that phage-encoded proteases might possibly activate some defence systems by proteolytic cleavage, yet further research is needed to confirm this hypothesis.

## 4. Viral effectors causing cellular stress

Viral infections induce significant stress on host cells that are able to trigger diverse defensive responses. In humans, a striking example comes from the dengue virus (DENV), which is an RNA virus that hides its genome by replicating inside semi-isolated membrane vesicles, thereby avoiding pattern receptor recognition [50]. These vesicles are virus-induced intracellular membrane rearrangements that lead to mitochondrial membrane damage and leakage of mitochondrial DNA (mtDNA). In turn, free mtDNA is sensed by the cGAS protein leading to activation of STING that induces an interferon response (figure 1) [51]. Certain viral proteins that perturb intracellular membranes have also been shown to activate the NLRP3 inflammasome, a key component of the innate immune response [52]. For instance, the M2 protein of influenza virus acts as an ion channel, pumping protons out of the Golgi lumen. This neutralizes the pH of the trans-Golgi network, creating an environment sufficient to activate the NLRP3 inflammasome [52]. Such findings suggest that a function of NLRP3 may be to monitor the cellular ion homeostasis and respond to its disruption within the cell.

A similar mechanism of membrane-disruption-induced immunity has been proposed in bacteria. During viral entry, phages typically induce membrane perturbations, or even employ enzymes on their tail fibres able to degrade extracellular polysaccharides to facilitate their access to the bacterial cell membrane [53]. By reducing the distance between the inner membrane and the peptidoglycan layer, these processes were suggested to participate in the activation of the Zorya defence system which is composed of four proteins (ZorA–D) [54]. ZorA and ZorB form an inner membrane-integrated complex (ZorA5_5_B2_2_) which senses the disruption of membrane integrity and transmits the signal to the effectors ZorC and ZorD, which bind and degrade invading phage DNA. The exact mechanism of phage-induced ZorAB anchoring and activation remains to be uncovered.

Another prominent example of stress induced by viral infection is DNA damage. In bacteria, this signal can be sensed by the Hachiman defence system [55]. Hachiman is composed of two proteins: the sensor helicase HamB detects free 3′-ssDNA ends resulting from phage-mediated DNA damage, upon which it activates the effector nuclease HamA, which indiscriminately degrades phage and host DNA within the cell to block infection (figure 1). Hachiman was initially shown to be activated by phage single-strand DNA-binding (SSB) proteins due to the emergence of phage SSB mutants that evade immunity [56]. However, latest findings demonstrate that drug-mediated DNA damage is sufficient to trigger Hachiman activity, suggesting that phage SSB proteins themselves induce DNA damage. Interestingly, other bacterial defence systems like Nhi and AbpAB are activated by phage SSB proteins, suggesting that these systems may sense DNA damage as well [57,58]. Eukaryotic viruses can also induce DNA damage during infection and an interplay was proposed between DNA damage repair and the activation of antiviral responses [59], together suggesting that DNA damage may be a shared virus-induced signal sensed by the immune system of prokaryotes and eukaryotes.

## 5. Viral immune evasion factors activate secondary immune responses

Viruses can evade immunity by mutating the viral component that is sensed by the host. However, the sensed components are often essential for viral propagation, which limits the scope of accessible mutations that enable immune evasion while maintaining viability. As a result, viruses have instead evolved a myriad of proteins that evade specific host immune systems [60–64]. Remarkably, distinct immune systems have in turn adapted to detect these viral suppressors directly or indirectly, thereby restoring immunity against escapers. Notably, such a defence strategy puts viruses in an evolutionary dilemma: those encoding evasion effectors might become sensitive to a second line of defence, while those lacking any would be targeted by a first line of defence.

A prominent example in humans involves the interaction between herpesviruses and the host restriction factor MORC3. Herpesviruses encode the protein ICP0, which facilitates the ubiquitylation and subsequent degradation of MORC3, thereby overcoming its antiviral activity [65]. However, MORC3 serves a dual role: beyond its function in viral restriction, it also acts as a repressor or ‘guard’ of interferon expression. The viral targeting of MORC3 by ICP0 therefore inadvertently triggers a secondary immune response. The virus-induced degradation of MORC3 relieves its repression of interferon, leading to the activation of a type I interferon response that initiates a broader, systemic line of defence (figure 1) [65]. While only a few such examples have been described in eukaryotic viruses, an increasing body of literature suggests that conceptually similar cases are prevalent in phage–bacteria interactions.

### (a) Phage-encoded anti-CRISPRs activate immune proteins

CRISPR-Cas systems, one of the most abundant families of defence systems in prokaryotes [66], were in some instances shown to transcriptionally control the expression of a second line of defence, thereby mirroring the MORC3-mediated de-repression of an interferon response (figure 1). To evade CRISPR-Cas immunity, phages have evolved multiple families of anti-CRISPR (Acr) proteins or small RNAs. Besides its activity as an RNA-guided nuclease, Cas9 was shown to also function as an auto-repressor of CRISPR-Cas expression, so that phage-encoded Acr proteins that disrupt Cas9 activity induce a burst in expression of the CRISPR-Cas locus during infection, thereby enhancing anti-phage defence [67]. Notably, certain CRISPR-Cas loci encode functional defence systems embedded between Cas genes, called CRISPR-Suppressed Immune Systems (CRISIS; figure 1). These embedded systems are repressed by the CRISPR-Cas complex under normal conditions and are thereby derepressed in the presence of Acr proteins, a mechanism proposed to provide a second line of defence when phages inhibit CRISPR-Cas [68]. Similarly, some CRISPR-Cas systems encode a CRISPR-repressed toxin whose expression is induced once the CRISPR-Cas complex is inhibited by Acr proteins or small RNAs, thereby leading to cell death or dormancy [69].

### (b) Phage-encoded inhibitors of restriction-modification systems induce cell death or dormancy

Beyond Acrs, phages also evolved anti-restriction proteins to evade restriction-modification (RM) systems. Among the first described examples is the 26-residue Stp protein from phage T4, which functions as an inhibitor of the RM system EcoprrI [70]. In addition to its three core subunits typical of type-I RM systems, EcoprrI has the particularity of encoding an additional protein called PrrC [71]. Under normal conditions, EcoprrI and PrrC form a stable complex and EcoprrI can cleave unmodified phage DNA like classical RM systems. However, binding and inhibition of EcoprrI by the phage Stp protein provokes a conformational change which activates PrrC into an anticodon nuclease cleaving lysine tRNAs, thereby shutting off translation [72].

Another notable case of phage immune evasion involves the Ocr protein from phage T7. Ocr is a DNA mimic, that is, a protein mimicking the structure and chemical properties of DNA [73], thereby acting as a decoy against defence systems that target phage DNA. Initially discovered as an inhibitor of RM and BREX defence systems [74,75], Ocr was more recently shown to trigger immune responses by unrelated defence systems. A remarkable example is PARIS (phage anti-restriction-induced system) [76]: PARIS is composed of two proteins, AriA and AriB, which form a stable complex in the absence of infection. Following Ocr binding to AriA during infection, a structural rearrangement releases AriB which acts as a nuclease cleaving lysine tRNAs to shut off translation [77,78]. Ocr being a decoy against RM and BREX systems, PARIS can therefore be seen as a decoy of a phage decoy. Another example of a defence system that senses anti-restriction proteins is Ronin: this system looks similar to an RM system that has lost its restriction component and instead encodes a short toxin gene called *ronA* [79]. Similarly to PrrC activation by Stp, anti-restriction proteins were proposed to be sensed by the modification subunits, leading to the activation of RonA to trigger cell death. PARIS and Ronin systems further illustrate the evolutionary dilemma encountered by such phages: phages that encode anti-restriction proteins would be sensitive to PARIS and Ronin, while phages lacking anti-restriction proteins would be targeted by RM and BREX systems.

### (c) Phage-encoded nucleic-acid-modifying proteins activate retron immunity

Phages can modify nucleic acids to evade bacterial defences such as RM systems, and they have evolved numerous strategies to do so. One prominent approach involves encoding DNA methylases that methylate viral DNA, rendering it unrecognizable to RM systems [80]. However, while these methylases help phages escape RM-mediated cleavage, they can inadvertently trigger other immune responses. For example, methylases can activate Dazbog and Nhi-like systems [56], though the exact molecular mechanisms remain unclear. Methylases can also activate a peculiar family of bacterial defence systems known as retrons. Retrons are atypical TA systems encoding a reverse transcriptase (RT), a non-coding RNA and a toxin [81–83]. Under basal conditions, the RT processes the non-coding RNA into a multi-copy single-stranded DNA/RNA hybrid (msDNA), yielding a stable tripartite complex comprising the RT, the msDNA and the inactive toxin. During phage infection, viral enzymes that inadvertently modify or degrade the msDNA unleash toxin activity, thereby aborting phage infection. For instance, a phage-encoded methylase modifies the msDNA of the Ec86 retron, leading to a conformational change and activation of the retron-associated toxin that depletes the essential metabolite NAD+ to block infection [84]. The phage-induced perturbations sensed by retrons are not limited to methylation: a phage-encoded exonuclease activates retron Sen2 through msDNA degradation [82], single-stranded DNA-binding proteins can activate retron Eco8 [56], while retrons Ec48 and Se72 can be activated by phage-encoded inhibitors of RecBCD [56,81], a bacterial exonuclease complex involved in DNA repair.

Altogether, increasing evidence shows that phage proteins that inhibit a first line of defence can be used as signals to activate a second line of defence, thereby providing a favourable immune strategy for the host. For instance, the RIIA-B proteins from phage T4 were initially discovered as inhibiting a bacterial defence system called RexAB [85]. RIIB was recently shown to activate a distinct defence system that forms pores in the bacterial membrane through the action of a bacterial gasdermin protein [10,86]. Another remarkable example is the phage anti-CBASS protein 2 (Acb2), a nucleotide ‘sponge’ that scavenges immune signalling molecules produced by bacterial CBASS systems. The Panoptes defence system evolved to sense such sponges through the action of a cyclase which constitutively produces decoy signalling molecules that inhibit an associated toxin; by sequestering these decoy molecules, Acb2 relieves toxin inhibition, thereby aborting infection [87,88]. The vast majority of bacterial defence systems known today were discovered in the past 5 years, and evasion proteins are yet to be described for over 80% of them [63]. We therefore anticipate that multiple evasion proteins will be discovered in the near future as activators of distinct defence systems.

## 6. Conclusion

The study of ETI across domains of life highlights a remarkable evolutionary strategy: the indirect sensing of pathogen activity. Monitoring the cellular state allows immune systems to observe the broader functional consequences of pathogen effector activity, thereby bypassing the need to coevolve with each individual effector of potentially diverse pathogens. Such efficiency underscores the evolutionary success of ETI as a defence strategy in plants, animals and bacteria alike. While our knowledge of ETI in plants is so far limited to bacterial pathogens, the propensity of viral effectors to trigger immunity in bacteria and animals suggests that virus-induced ETI exists in plants as well. Furthermore, the evolutionary parallels between strategies encoded by prokaryotic and eukaryotic viruses provide a unifying framework for studying host–pathogen interactions. Both eukaryotic viruses and phages deploy evasion proteins to counteract host defences, sometimes targeting ancestral immune pathways that are common to prokaryotes and eukaryotes. This convergence suggests that insights gained from one viral domain can inform our understanding of the other, bridging the study of prokaryotic and eukaryotic virology. For example, structural analyses of animal poxvirus proteins that inhibit cGAS-STING signalling revealed striking homology with phage-encoded Acb1 proteins, which target the CBASS bacterial defence system [89]. Comparative structures of poxvirus protein-2′3′-cGAMP and phage Acb1-3′3′-cGAMP complexes demonstrated a universal mechanism for degrading nucleotide-based immune signals, while highlighting adaptations specific to each domain [90]. Since multiple evolutionary conserved immune components were recently discovered (Box 1), we anticipate that the similarities in the immune evasion strategies employed by prokaryotic and eukaryotic viruses go far beyond current knowledge.

Looking forward, the continued investigation of viral effectors and their interactions with host immune systems promises to reveal novel immune mechanisms and deepen our understanding of host–pathogen interactions and their evolutionary dynamics. By focusing on the commonalities across domains of life, we can develop a more integrated view of immunity, one that encompasses the shared strategies that have emerged in response to the persistent arms race between hosts and their pathogens.

## Data Availability

This article has no additional data.
